# Live‐cell imaging of rice cytological changes reveals the importance of host vacuole maintenance for biotrophic invasion by blast fungus, *Magnaporthe oryzae*

**DOI:** 10.1002/mbo3.304

**Published:** 2015-10-15

**Authors:** Susumu Mochizuki, Eiichi Minami, Yoko Nishizawa

**Affiliations:** ^1^ Genetically Modified Organism Research CenterNational Institute of Agrobiological SciencesKannondai 2‐1‐2TsukubaIbaraki305‐8602Japan; ^2^ Graduate School and Faculty of AgricultureKagawa UniversityMikiKagawa761‐0795Japan

**Keywords:** BIC structure, long‐term live‐cell fluorescence imaging method, *Magnaporthe oryzae*, rice blast fungus, vacuole.

## Abstract

The rice blast fungus *Magnaporthe oryzae* grows inside living host cells. Cytological analyses by live‐cell imaging have revealed characteristics of the biotrophic invasion, particularly the extrainvasive hyphal membrane (EIHM) originating from the host plasma membrane and a host membrane‐rich structure, biotrophic interfacial complex (BIC). Here, we observed rice subcellular changes associated with invasive hyphal growth using various transformants expressing specifically localized fluorescent proteins. The invasive hyphae did not penetrate across but were surrounded by the host vacuolar membrane together with EIHM even after branching. High‐resolution imaging of BICs revealed that the host cytosol was accumulated at BIC with aggregated EIHM and a symplastic effector, Pwl2, in a punctate form. The vacuolar membrane did not aggregate in but closely surrounded the BIC. A good correlation was observed between the early collapse of vacuoles and damage of invasive hyphae in the first‐invaded cell. Furthermore, a newly developed, long‐term imaging method has revealed that the central vacuole gradually shrank until collapse, which was caused by the hyphal invasion occurring earlier in the neighboring cells than in the first‐invaded cells. These data suggest that *M. oryzae* may suppress host vacuole collapse during early infection stages for successful infection.

## Introduction

Rice blast caused by the ascomycete fungus, *Magnaporthe oryzae*, is the most destructive disease affecting cultivated rice (*Oryza sativa* L.). Infection by *M. oryzae* leads to annual yield losses of 10–30% (Skamnioti and Gurr 2009). Because genetic manipulation techniques and whole genome sequences are available for both *M. oryzae* and rice, the *M. oryzae*–rice pathosystem is a good model for investigating plant diseases caused by filamentous pathogens (Wilson and Talbot 2009).

Extensive microscopic studies have revealed the foliar infection process and hemibiotrophic behavior of *M. oryzae*. The spore that attaches to the hydrophobic cuticle of rice cells germinates at 0.5–1.5 h post inoculation (hpi) and subsequently produces an appressorium, which is a dome‐shaped infection‐specific structure at the tip of the germ tube (2–8* *hpi). The appressorium melanizes and develops substantial turgor, which translates into physical force to puncture the cuticle (de Jong et al. 1997). A narrow penetration peg forms at the base of the appressorium and enters rice epidermal cells utilizing the turgor (16–20* *hpi). The penetration peg subsequently differentiates into infectious hyphae, which are primarily thin, then become bulbous, and branch (20–36* *hpi). At this stage, the invaded cells of the susceptible host plasmolyze in a hypertonic solution, indicating that they remain alive, whereas in the resistant host, the invaded cells lose the ability for plasmolysis and exhibit strong autofluorescence, indicating hypersensitive cell death (Koga 1994). The bulbous invasive hyphae usually grow to fill the first‐invaded cell and then colonize neighboring cells (36–48* *hpi). At this stage, the first‐invaded cells lose the ability for plasmolysis, whereas the second‐invaded cells keep the ability. The plasmolysis experiments indicated that *M. oryzae* sequentially invade living host cells (Koga et al. 2004; Kankanala et al. 2007). Finally, lesions become visible (ca. 72 hpi), and sporulation occurs under humid conditions.

Cytological analysis by live‐cell imaging using a confocal laser scanning fluorescence microscope has provided new insights into the events occurring during a biotrophic interaction between *M. oryzae* and rice. Invasive hyphae are sealed in a host membrane, termed the extrainvasive hyphal membrane (EIHM) (Kankanala et al. 2007), originating in the host plasma membrane (Mentlak et al. 2012). EIHM forms a membrane cap at the tip of the primary hyphae, which is later subapically positioned as bulbous invasive hyphae develop within the first‐invaded cells. The novel membrane‐rich in planta structure is named the biotrophic interfacial complex (BIC) (Khang et al. 2010), and host endoplasmic reticulum (ER) accumulates around BIC (Giraldo et al. 2013). Time‐lapse imaging has shown that invasive hyphae possibly scan plant cell walls before crossing and transmission electron microscopy has shown invasive hyphae preferentially crossing cell walls at pit fields, the area where the plasmodesmata concentrate (Kankanala et al. 2007). When invasive hyphae move into neighboring cells, the plasma membranes of the second‐invaded cells invaginate again to surround the growing hyphae, and the BIC structure initially appears adjacent to primary hyphal tips, then subapically positions (Kankanala et al. 2007; Khang et al. 2010).

Several effector candidates were defined as biotrophy‐associated secreted (BAS) proteins, which demonstrate distinct patterns of accumulation within the host tissue during the biotrophic invasion (Mosquera et al. 2009). Apoplastic effectors, which do not enter host cells, are generally dispersed and retained in the matrix between the fungal cell walls and EIHM (extrainvasive hyphal matrix; EIHMx); thus, they outline the entire invasive hyphae uniformly during the biotrophic invasion. In contrast, symplastic effectors, which move into host cells, preferentially accumulate in BIC. Moreover, the BIC‐associated initial bulbous cell is enriched in secretion machinery components for symplastic effectors; thus, BIC is predicted to be involved in the delivery of symplastic effectors (Mosquera et al. 2009; Khang et al. 2010; Giraldo et al. 2013). These extensive investigations on live‐cell fluorescence imaging of infected leaf tissues provided an important framework of cytological characteristics of the biotrophic invasion: BIC and EIHM.

Plant cells have a large central vacuole that accumulates various hydrolytic enzymes and antimicrobial compounds, suggesting that vacuoles play a role in plant immunity. Two vacuole‐mediated plant defense strategies associated with hypersensitive cell death were proposed, which are (1) disruption of the vacuolar membrane mediated by the vacuolar processing enzyme, releasing vacuolar contents into the cytoplasm in response to viral infection (Hatsugai et al. 2004) and (2) proteasome‐dependent fusion of the vacuole with the plasma membrane, discharging vacuolar contents from the cell in response to bacterial infection (Hatsugai et al. 2009). However, the involvement of the vacuole in response to filamentous pathogens is unknown. Live‐cell imaging of vacuolar membranes during infection has been reported in Arabidopsis (*Arabidopsis thaliana*). The vacuolar membrane is located close to the extrahaustorial membrane (EHM) surrounding the haustorium of the powdery mildew fungi *Erysiphe cichoracearum* (Koh et al. 2005) and the downy mildew oomycete *Hyaloperonospora arabidopsidis* (Caillaud et al. 2012). However, insight into vacuoles in the infected rice cells is lacking.

In this study, we observed the host subcellular changes, particularly the dynamics of vacuolar membranes and the BIC structure associated with the growth of *M. oryzae* using transformants expressing fluorescent protein. To simultaneously and sequentially monitor growing invasive hyphae and host organelle, we developed a long‐term live‐cell fluorescence imaging method using a high‐speed confocal laser scanning system. We demonstrated that invasive hyphae invaginate vacuolar membranes and are closely surrounded by the membranes. A good correlation was observed between the early collapse of vacuoles and damage of invasive hyphae in the first‐invaded cell. In contrast, the long‐term sequential imaging showed that the early collapse of the vacuole did not terminate the hyphal growth in the subsequently invaded cells. These results indicate that in the compatible interaction, the host vacuole is maintained until a certain stage of fungal development, which may be important for successful infection by *M. oryzae*.

## Experimental Procedures

### Plant materials, transformation, and growth condition

Rice seeds (*O. sativa* L. japonica cv. Nipponbare BL no. 2 harboring *Pii* and *Pish* and BL no. 5 harboring *Pik‐* and *Pish*‐resistant genes against *M*. *oryzae*) were supplied by Dr. H. Satoh of the National Institute of Crop Science, Tsukuba, Japan. We produced transgenic rice lines as shown in Table 1. Rice transformation was performed according to Toki et al. (2006). At least three transgenic lines were selected for each vector construct based on fluorescence intensity, localization, and growth normality. For inoculation, husked and surface‐sterilized seeds were sown onto 1% (w/v) agar, which contained quarter‐strength Murashige–Skoog's medium, 25 mg L^−1^ hygromycin (Wako Pure Chemical, Osaka, Japan) and 6.25 mg L^−1^ meropen (Sumitomo Dainippon Pharma, Osaka, Japan). Plants were grown for 4 days on the agar medium at 22°C under continuous light (35 *μ*mol m^−2^ sec^−1^) and then grown hydroponically for 3–4 weeks with a nutrient solution as described previously (Tanabe et al. 2006) in a growth chamber with a 14‐h light (28°C)/10‐h dark (25°C) cycle. Localization of green fluorescent protein (GFP) signals in leaf sheath epidermal cells of each transgenic line was confirmed by fluorescence microscopy (Fig. S10).

**Table 1:** Transformants of rice and *Magnaporthe oryzae* used in this work

Line	Promoter	Protein	Target	Reference
Rice				
cyto‐GFP	EN4	AcGFP1	Cytosol	This work
er‐GFP	35S	EGFP:HDEL	Endoplasmic reticulum	Haseloff et al. (1997)
vm‐GFP	EN4	HvSUT2:AcGFP1	Vacuolar membrane	Endler et al. (2006)
pm‐GFP	35S	EGFP:LTi6b	Plasma membrane	Cutler et al. (2000)
				Kurup et al. (2005)
*Magnaporthe oryzae*				
TmC	TEF	mCherry	Cytosol	Vanden Wymelenberg et al. (1997)
				Kouzai et al. (2014)
BAS4mC	BAS4	Bas4:mCherry	EIHMx	Mosquera et al. (2009)
PWL2mC	PWL2	Pwl2:mCherry	BIC	Khang et al. (2010)
ProG+PWL2mC	PWL2	AcGFP1	Expression & localization of Pwl2	This work
	PWL2	Pwl2:mCherry		

### Fungal materials, transformation, and culture condition

We used transformants (Table 1), which were generated using *M. oryzae* strains Ina86–137 (race 007.1; MAFF Gene Bank stock number MAFF101511) and P91–15B (race 001.1; provided by Prof. H. Naitoh of Akita Prefectural University), Japanese pathogenic isolates from rice plants. *Agrobacterium*‐mediated transformation of *M. oryzae* was performed according to Saitoh et al. (2008). At least three transformants were selected for each vector construct, based on fluorescence intensity, growth, and conidiation on the media plate, and virulence. To prepare conidia, fungal strains were cultured on oatmeal agar medium (5% oatmeal powder, 2% sucrose, 1.5% agar) for 7 days at 25°C in darkness. Conidial production was induced by irradiation with a blacklight blue lamp for 4 days after the aerial mycelia were removed using a sterilized brush.

### Plasmids

For the production of cyto‐GFP lines, an *AcGFP1* fragment was amplified by PCR from pAcGFP1‐Hyg‐N1 (Clontech, Palo Alto, CA) and cloned under the enhanced 35S promoter (EN4) in pBI333‐EN4 (Nishizawa et al. 1999). For er‐GFP lines, an *EGFP:HDEL* fragment was PCR amplified from pCB302, kindly provided by Dr. B. Müller of Zürich University, and cloned under the Cauliflower Mosaic Virus (CaMV) 35S promoter in pBI333 using InFusion technology (Clontech). For vacuolar membranes GFP (vm‐GFP) lines, a cDNA fragment for *HvSUT2* (Endler et al. 2006) was isolated from total RNA prepared from seedlings of *Hordeum vulgare* cv. Golden Promise and inserted under the EN4 promoter in pBI333‐EN4 with an *AcGFP1* fragment at the C‐terminal. For pm‐GFP lines, pBIB‐35S‐EGFP‐LTi6b, kindly provided by Dr. S. Kurup of University of Cambridge, was used as is.

TmC lines were produced using pCAMBIA‐TCT (Kouzai et al. 2014). Effector:mCherry expression plasmids for generating BAS4mC and PWL2mC lines were constructed as described below. The *trpC* terminator was PCR amplified from pCSN43 (Staben et al. 1989) and cloned into pENTR (Thermo Fisher Scientific Inc., Waltham, MA) with *mCherry* (Clontech) for generating pEmCT. DNA fragments, including the promoter and coding regions without the stop codon for *BAS4* (Mosquera et al. 2009) and *PWL2* (Khang et al. 2010), were PCR amplified from genomic DNA of Guy11 (supplied by Dr. M. Nishimura of NIAS, Tsukuba, Japan) and Ina86‐137, respectively. Furthermore, the amplified fragments were cloned into pEmCT for generating pE‐BAS4full‐mCT and pE‐PWL2full‐mCT, respectively. These entry vectors and a destination vector, pCAMBIA‐Bar‐RfA (Saitoh et al. 2008), were treated with LR clonase in Gateway cloning kit (Thermo Fisher Scientific Inc.) for generating pCAMBIA‐Bar‐BAS4full‐mCT and pCAMBIA‐Bar‐PWL2full‐mCT, respectively. For the production of the ProG + PWL2mC lines, an *AcGFP1* fragment was amplified by PCR from pAcGFP1‐Hyg‐N1 (Clontech) using AcGFP5′forIF and AcGFP3′forIF primers and replaced mCherry in pEmCT using the InFusion technology (Clontech). The resultant pE‐AcGT was fused with the *PWL2* promoter region (ca. 0.6 kb) that was PCR amplified using Ppwl2‐5′forIF and Ppwl2‐3′forIF primers by InFusion for generating pE‐Ppwl2‐AcGFPT. Furthermore, the fragment for PWL2full‐mCherry was PCR amplified from pE‐PWL2full‐mCT using PWL2‐5′forIF(S) and mC‐3′forIF primers and performed InFusion with pE‐Ppwl2‐AcGFPT for generating pE‐PWL2full‐mC‐Ppwl2‐AcGT. This entry vector and pCAMBIA‐Bar‐RfA were fused using the Gateway cloning system (Thermo Fisher Scientific, Inc.) for generating pCAMBIA‐Bar‐PWL2full‐mC‐Ppwl2‐AcGT. Primer sets used for PCR amplification were shown in Table S1.

### Fluorescence imaging at a fixed time point

Leaf sheaths, which are cylindrical, from the sixth leaves of the seven‐leaf stage plants were excised, filled with conidia suspension (1 × 10^5^ conidia mL^−1^) using a syringe, and incubated for 24, 30, 36, or 42 h at 25°C in the dark. Three cm of the inoculated leaf sheaths were excised for preparing the sliced inner epidermis using a thin knife and scissors (Fig. S6A) and mounted onto a slide glass with water. GFP and mCherry fluorescence was observed using an epifluorescence microscope (DM6000B; Leica Microsystems Inc., Wetzlar, Germany) equipped with a confocal laser scanning unit (CSU‐X1; Yokogawa Electric, Tokyo, Japan), the laser units (Sapphire 488 and 561 nm; Coherent, Santa Clara, CA), dichroic mirror (DM‐405/488/561), and emission filters (GFP, EM‐520/35; mCherry, EM617/73). Fluorescence images were acquired using an EM‐CCD camera (iXon897; Andor Technology PLC., Belfast, Northern Ireland, U.K.) with a 63× glycerol immersion objective (Leica Microsystems Inc.). The magnified fluorescence images shown in Figure 4 and Figures S5, S7 were acquired under the confocal fluorescence microscopy system TCS‐SP5 (Leica Microsystems Inc.) (GFP, ex. 488 nm/em. 500–520 nm; mCherry, ex. 561 nm/580–600 nm). Images were analyzed using MetaMorph software (Molecular devices LLC, Sunnyvale, CA) and LAS‐AF software (Leica Microsystems Inc.).

### Time‐lapse fluorescence imaging

A 3‐mm thick silicone spacer (1.5 × 4.0 cm) was placed on a glass slide and 1% agarose (UltraPure low‐melting point agarose [Thermo Fisher Scientific Inc.] in phosphate‐buffered saline PBS] [pH 7.4]) was poured into a space surrounded by the silicone spacer (Fig. S6B). Sliced leaf sheath inner epidermis (3‐cm long) were prepared from the sixth leaves (Fig. S6A) and kept in water for 2 h. The sliced tissues were mounted on the agarose before the agarose completely solidified and incubated in dark in a moist case for 1 h. Subsequently, 200 *μ*L of conidial suspension (5 × 10^5^ conidia mL^−1^) was placed on the sliced tissues and incubated at 25°C in the dark for 12 h in a moist case. After the successful fungal penetration into the rice cells was microscopically confirmed, the samples were filled with 1 mL silicone oil (dimethylpolysiloxane [200 cSt; Sigma–Aldrich, St. Louis, MO]) and carefully covered with a coverslip. GFP and mCherry fluorescence was observed using a confocal laser scanning microscopy described in the previous section and installed in the room at 25°C. Fluorescence images were acquired at 20‐ or 30‐min intervals using an EM‐CCD camera (iXon897; Andor Technology PLC.) with a 20× long working distance objective (Leica Microsystems Inc.). The *z*‐series of optical sections corresponding to the outer half of the inner epidermal cells were stacked for generating maximum‐intensity projection images. Each maximum‐projection image was connected to produce motion pictures. Imaging data were processed using MetaMorph software (Molecular devices LLC) and QuickTime (ver. 7; Apple Inc., Cupertino, CA).

## Results

### Invasive hyphae grow inside the rice cell surrounded by the vacuolar membrane

**Figure 1:**
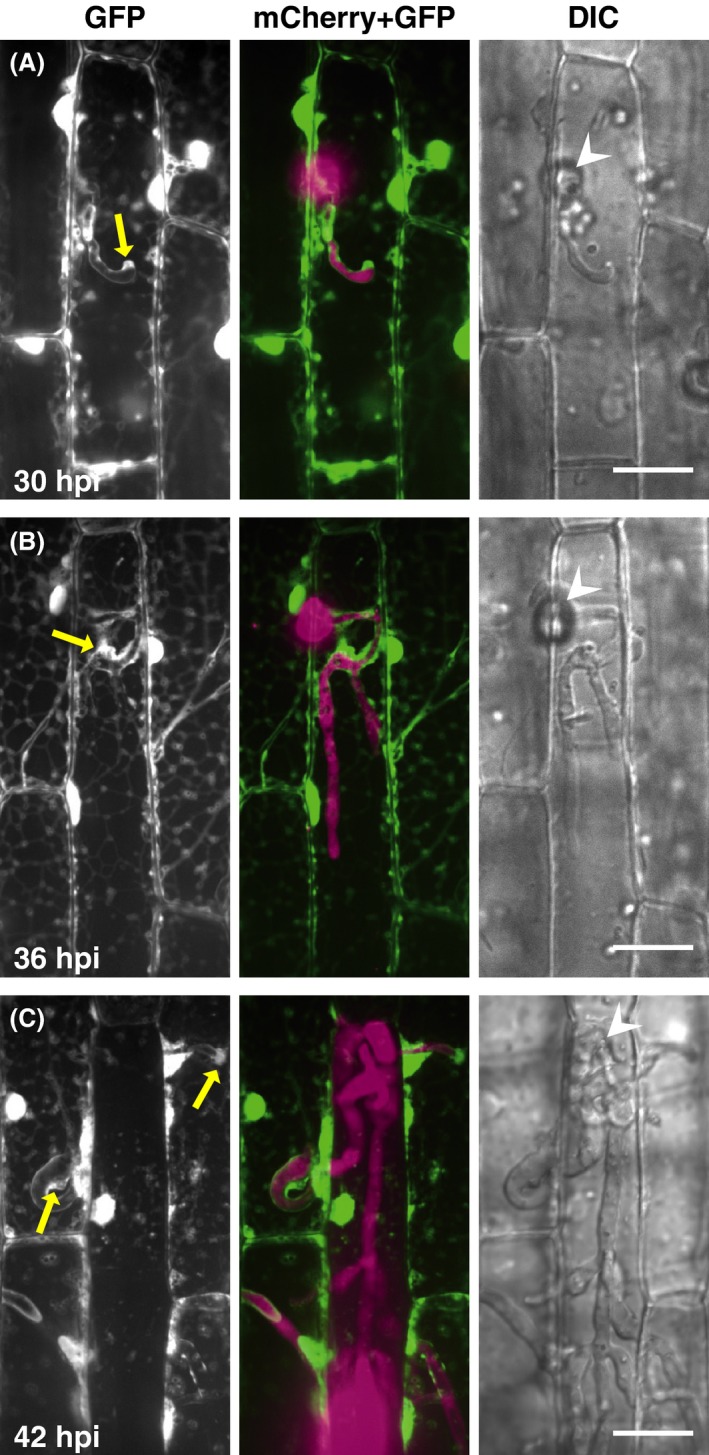
Visualization of host cytosol during *Magnaporthe oryzae* infections. Leaf sheaths of transgenic rice plants constitutively expressing AcGFP1 (cyto‐GFP line) were inoculated with a conidial suspension of a compatible strain (Ina86‐137) transformed with *tefp::mCherry* (TmC1 line) and observed using a laser confocal microscope. Representative data of the 8 (A), 15 (B), and 12 (C) similar images are shown. Cyto‐GFP signals are detected along the primary invasive hyphae both in the first‐invaded (A and B) and second‐invaded cells (C), in addition to the predicted positions of the BIC (yellow arrows). GFP, stacked *z*‐series confocal fluorescence images of GFP signals corresponding roughly to the surface half of rice epidermal cells. mCherry + GFP, mergers of the GFP image, and stacked *z*‐series confocal fluorescence images of mCherry signals. Wedge, appressorial penetration site. Size bar = 20 *μ*m. BIC, biotrophic interfacial complex; GFP, green fluorescent protein; DIC, differential interference contrast images.

We first investigated the dynamics of rice cytosol in leaf sheath epidermal cells during the biotrophic invasion by *M. oryzae*. We used transgenic rice plants expressing the GFP in the cytosol (cyto‐GFP line) and a virulent strain constitutively expressing a red fluorescent protein, mCherry, in the fungal cytosol (TmC line) (Table 1). During the early stage when BIC was predicted to be located at the primary hyphal tip (Fig. 1A), Cyto‐GFP signals were accumulated near the fungal penetration site beneath the appressorium and at the primary hyphal tip in addition to the host nucleus. This was consistent with the observations using an optical microscope of cytoplasm accumulated beneath the appressorial penetration site and around BIC (Khang et al. 2010). Cyto‐GFP was also observed to outline the primary hyphae. At the stage when the presumed BIC was left behind the extending hyphae (Fig. 1B), cyto‐GFP was detected at the presumed BIC and along the primary hyphae. Furthermore, cyto‐GFP associated with the growing bulbous invasive hyphae was faint and not detected along the anterior half of those hyphae. At the stage when invasive hyphae penetrated into the neighboring cells (Fig. 1C), strong GFP signals were detected at the penetration area in the second‐invaded cells, often accumulated in a delta shape at the base of the penetrating hyphae. Cyto‐GFP was also detected at the hyphal tips, which presumably corresponded to BIC and once again outlined invasive hyphae. These cytosolic patterns were consistent with the fluorescence signals observed in the invaded cells labeled with the ER‐localized GFP (er‐GFP) (Fig. S1).

**Figure 2:**
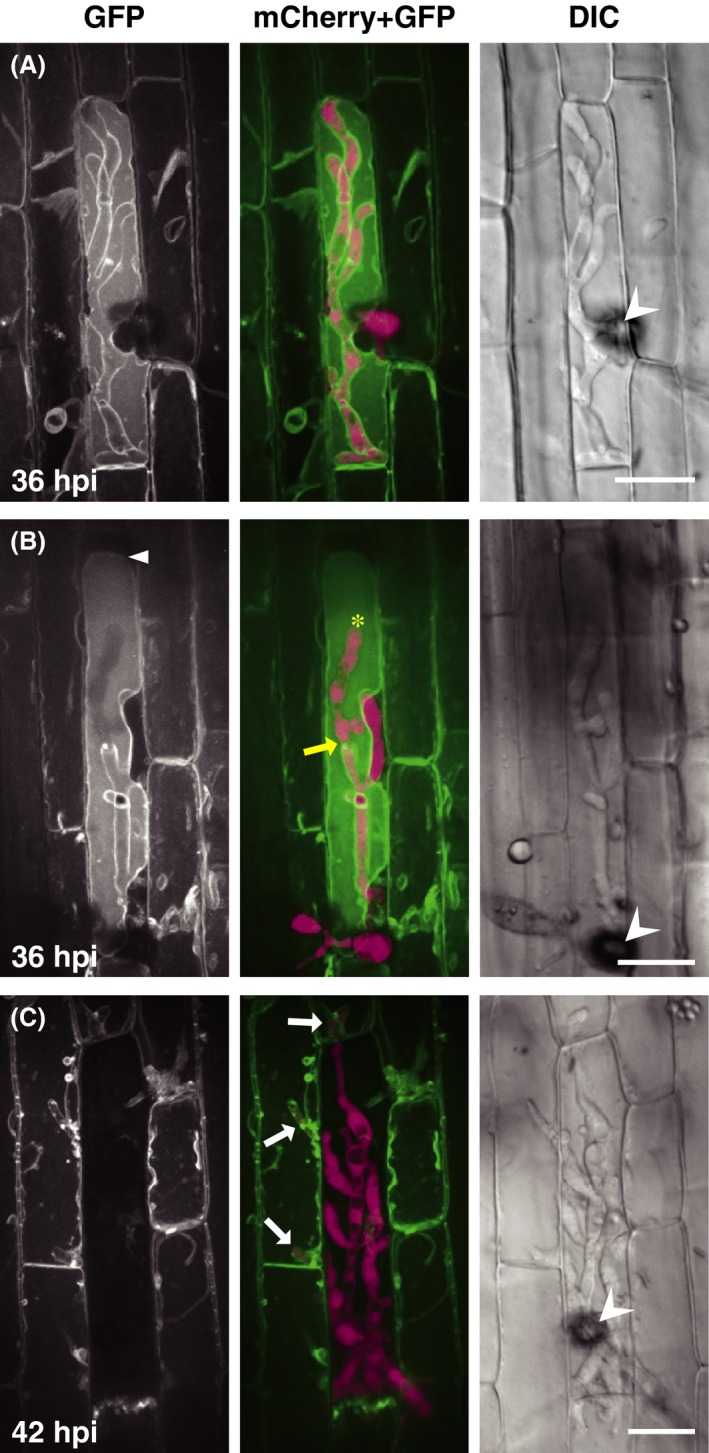
Invasive hyphae of *Magnaporthe oryzae* are surrounded by host vacuolar membranes. Leaf sheaths of transgenic rice plants constitutively expressing HvSUT2:AcGFP1 (vm‐GFP line) were inoculated with a conidial suspension of the compatible TmC line and observed using a laser confocal microscope. Invasive hyphae are outlined by vm‐GFP signals both in the primary‐ (A and B) and secondary‐invaded cells (C; white arrows). Vacuolar shrinkage is often observed (white arrowhead in B). Representative data of 5 (A), 6 (B), and 19 (C) similar images are shown. Asterisk and yellow arrow in the B (mCherry + GFP) panel indicate an invasive hypha inside the vacuole and the predicted point of penetrating across the vacuolar membrane, respectively. GFP, stacked *z*‐series confocal fluorescence images. mCherry + GFP, mergers of the GFP image, and stacked *z*‐series confocal fluorescence images of mCherry signals. Wedge, appressorium. Size bar = 20 *μ*m. GFP, green fluorescent protein; DIC, differential interference contrast images.

Thin layers of cyto‐GFP outlining the bulbous invasive hyphae suggested that the hyphae were tightly surrounded by the host central vacuole. We next observed rice vacuolar membranes in leaf sheath epidermal cells invaded by the TmC line. The rice vacuolar membrane was labeled with GFP fused with a barley sucrose transporter (Endler et al. 2006). At the stage of single cell invasion, the GFP signals (vm‐GFP) outlined the highly branching bulbous invasive hyphae (Fig. 2A), indicating that the invasive hyphae did not penetrate across but were surrounded by the host vacuolar membranes. Vacuole shrinking was often observed at the stage when the invasive hyphae developed to branch (the white arrowhead in Fig. 2B). Figure 2B also shows the invasive hyphae that grew inside the vacuole as indicated by the asterisk. In the subsequently invaded cells, invasive hyphae were again surrounded by vm‐GFP (Fig. 2C).

We also observed intracellular structures in rice cells invaded by an avirulent strain. When a resistant rice cultivar expressing cyto‐GFP, er‐GFP, or vm‐GFP was inoculated with the TmC line, GFP signals in invaded cells disappeared before the hyphae developed to branch, indicating hypersensitive cell death (data not shown). However, GFP signals outlined as yet short invasive hyphae in some infection sites (Fig. S2), indicating that invasive hyphae of the avirulent strain are also encompassed by the host vacuole at the early infection stage.

**Figure 3:**
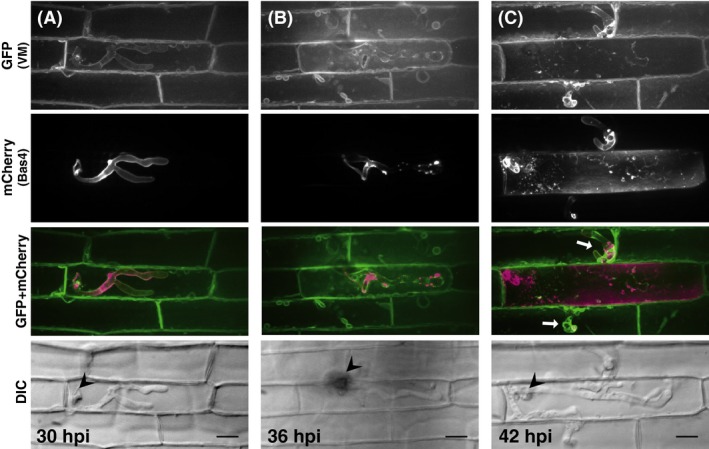
Dual observation of host vacuolar membranes and EIHM during *Magnaporthe oryzae* infections. Leaf sheaths of vm‐GFP rice were inoculated with a conidial suspension of a compatible strain (Ina86‐137) transformed with *BAS4p::BAS4:mCherry* (BAS4mC line) and observed using a laser confocal microscope. Representative data of 26 (A), 15 (B), and 18 (C) similar images are shown. Smooth outlining of invasive hyphae with Bas4:mCherry signals indicates the integrity of EIHM. When vm‐GFP signals smoothly outline the invasive hyphae (A and white arrows in C), EIHM integrity remains. In contrast, when the contours of hyphae outlined by vm‐GFP are unclear, Bas4:mCherry signals are patchy (B). GFP (VM) and mCherry(Bas4), stacked *z*‐series confocal fluorescence images of GFP, and mCherry signals, respectively. GFP + mCherry, mergers of GFP, and mCherry images. Wedge, appressorial penetration site. Size bar = 20 *μ*m. EIHM, extrainvasive hyphal membrane; GFP, green fluorescent protein; VM, vacuolar membranes; DIC, differential interference contrast images.

Cyto‐GFP and er‐GFP signals were no longer detected along the extended bulbous hyphae (Fig. 1B; Fig. S1A), whereas vm‐GFP continued to outline highly branching bulbous hyphae (Fig. 2A). These observations suggested that a large part of the extended bulbous hyphae was tightly encased by both the vacuolar membrane and EIHM if it remained. To analyze the association between the vacuolar membrane and EIHM, we inoculated rice leaf sheaths expressing vm‐GFP with a transformant expressing Bas4:mCherry. Bas4 accumulates in EIHMx after being secreted from the fungus. Thus, Bas4:mCherry indirectly visualizes EIHM (Mosquera et al. 2009). Both vm‐GFP and Bas4:mCherry were closely located outlining the primary hyphae and bulbous invasive hyphae (Fig. 3A), indicating that the hyphae were doubly encased by the vacuolar membrane and EIHM. When the contours of hyphae outlined by vm‐GFP were unclear (Fig. 3B) or the vacuole shrank and disappeared (Fig. S3), Bas4:mCherry signals were patchy, suggesting that EIHM lost integrity. When the invasive hyphae penetrated into the neighboring cells (Fig. 3C), they were again encased by the vacuolar membrane and EIHM, while both signals of vm‐GFP and Bas4:mCherry scattered in the first‐invaded cells.

### BIC includes host cytosol and punctate effectors, but not the vacuolar membrane

**Figure 4:**
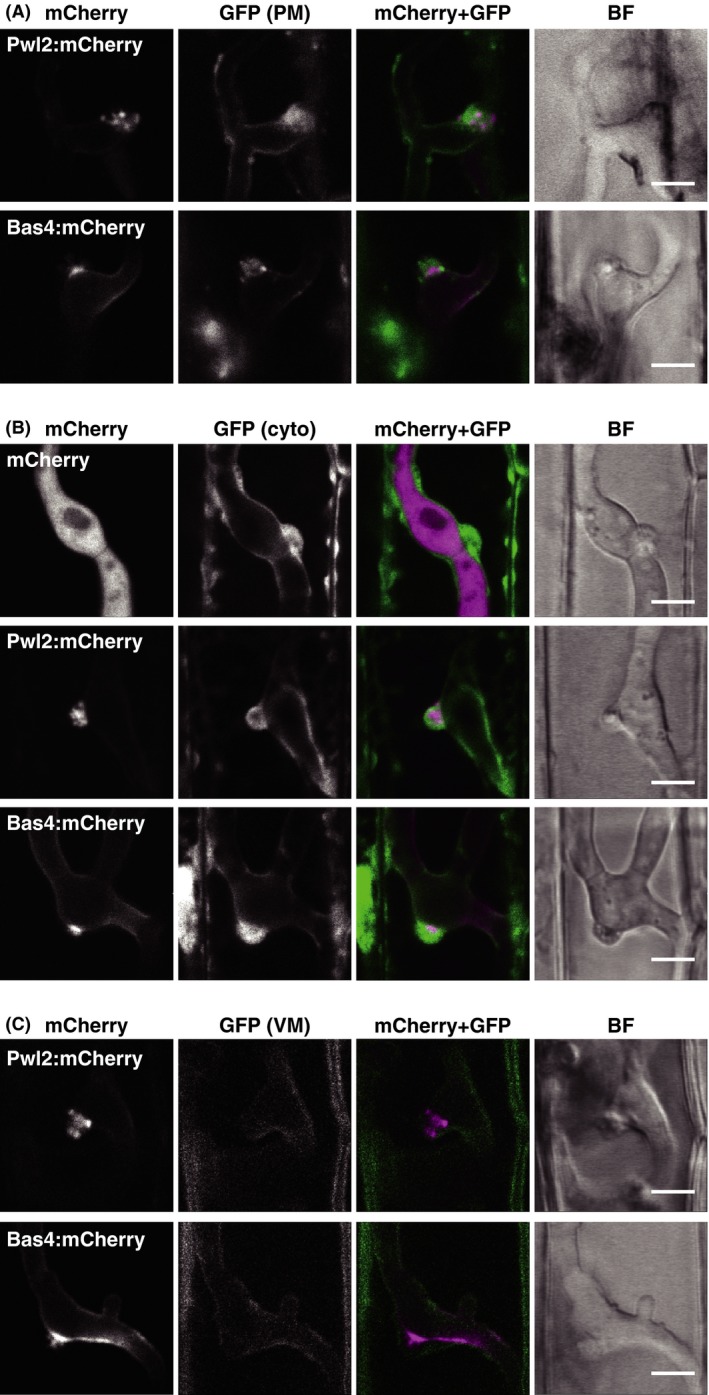
Confocal micrographs of the BIC. (A) Aggregation of the host plasma membrane‐derived at BIC. Leaf sheaths of transgenic rice plants constitutively expressing EGFP:LTi6b (pm‐GFP line) were inoculated with a conidial suspension of a compatible strain (Ina86‐137) transformed with *PWL2p::PWL2:mCherry* (PWL2mC line) or BAS4mC line. Representative data of 13 (PWL2mC) and 9 (BAS4mC) similar images are shown. (B) Accumulation of host cytosol at the BIC. Leaf sheaths of cyto‐GFP rice were inoculated with the compatible TmC, PWL2mC, or BAS4mC line. Representative data of 11 (TmC), 24 (PWL2mC), and 8 (BAS4mC) similar images are shown. (C) Surrounding of the BIC by host vacuolar membrane. Leaf sheaths of vm‐GFP rice were inoculated with the compatible PWL2mC or BAS4mC line. Representative data of 15 (PWL2mC) and 7 (BAS4mC) similar images are shown. These observations also reveal that Pwl2:mCherry is localized in BICs as puncta. All images are the single optical sections captured at 30 hpi. Size bar = 5 *μ*m. BIC, biotrophic interfacial complex; GFP, green fluorescent protein; BF, bright field image.

To characterize the BIC structure and clarify the association with the vacuolar membrane, we observed BICs that formed in rice cells labeled with plasma membrane‐localized GFP (pm‐GFP), cyto‐GFP, or vm‐GFP at high resolution (Fig. 4). Rice plasma membrane was fluorescently labeled using GFP‐LTi6b (Cutler et al. 2000) according to Mentlak et al. (2012). BICs were visualized using a fluorescently labeled symplastic effector, Pwl2:mCherry according to Khang et al. (2010). An apoplastic effector, Bas4, exhibits partial colocalization with Pwl2 in BICs in addition to the uniform envelopment of growing hyphae (Mosquera et al. 2009); thus, we also inoculated each transgenic rice line with a blast strain expressing Bas4:mCherry to simultaneously visualize BIC positions and EIHM. First, we confirmed that pm‐GFP signals outlined invasive hyphae, namely visualized EIHM, in our experimental conditions (Fig. S4). As shown in Figure 4A, pm‐GFP aggregated around Pwl2:mCherry and Bas4:mCherry accumulating sites to form the BIC structure. Cyto‐GFP also concentrated at BICs in a dome shape (Fig. 4B), consistent with the observations shown in Figure 1. These results demonstrated that the BIC is not only a membrane‐rich, but also cytosol‐rich structure. On the other hand, vm‐GFP outlined BICs and hyphae, but not accumulated at the BIC (Fig. 4C).

Fluorescence imaging of BICs with high resolution also revealed that Pwl2:mCherry was detected in BICs as puncta (Fig. 4; Fig. S5). The diameter of bright puncta was ca. 500 nm. In contrast, Bas4:mCherry was hardly detected as puncta at BICs. Bas4:mCherry accumulated only in the periphery of the BIC‐associated cell at the base of BIC (Fig. 4), which was consistent with the fact that Bas4 is an apoplastic effector (Mosquera et al. 2009). The space between vm‐GFP and Bas4:mCherry signals at BIC represented the region where Bas4 did not reach (Fig. 4C). Altogether, these results indicated that BIC contains the host cytosol and a symplastic effector in a punctate form in addition to the host plasma membrane‐derived membrane, and the central vacuolar membrane surrounds the BIC structure, but not intermingles with the accumulated plasma membrane/EIHM.

### Host vacuole shrinks as hyphae grow

The vacuole is a dynamic organelle, constantly changing its morphology (Uemura et al. 2002). As described earlier, vacuole shrinking was often observed in the first‐invaded cells, and the vacuole ultimately collapsed (Fig. 2B and C). To clarify whether the vacuole shrinking observed in invaded cells is just temporary or halfway to the collapse associated with the growth of invasive hyphae, we introduced a newly developed imaging method that enables simultaneous recording of both growing hyphae and host intracellular structures sequentially for over 30 h. Hand‐sliced inner epidermis of leaf sheaths was mounted on agarose gel set on a slide glass and kept 3 h before inoculation for attenuating wound responses, including autofluorescence. The inoculated tissues were incubated in a moisturized box until appressoria were differentiated, and then filled with silicone oil and covered with a coverslip (Fig. S6). It was important to keep the inoculated tissues uncovered until the stage of appressorial formation and to subsequently cover tissues using the silicone oil dimethylpolysiloxane 200 cSt. Failure of these conditions led to the termination of invasion with high frequency. To minimize the exposure to laser beams that lead to the inactivation of plant and fungal cells and the bleaching of fluorescence signals, we used a high‐speed confocal laser scanning apparatus and a super high‐sensitivity camera and recorded images at 20‐ or 30‐min intervals. This time‐lapse imaging system sequentially captured infection process from the stage before appressorial maturation to the stage of colonizing neighboring cells (Movie S1).

**Figure 5:**
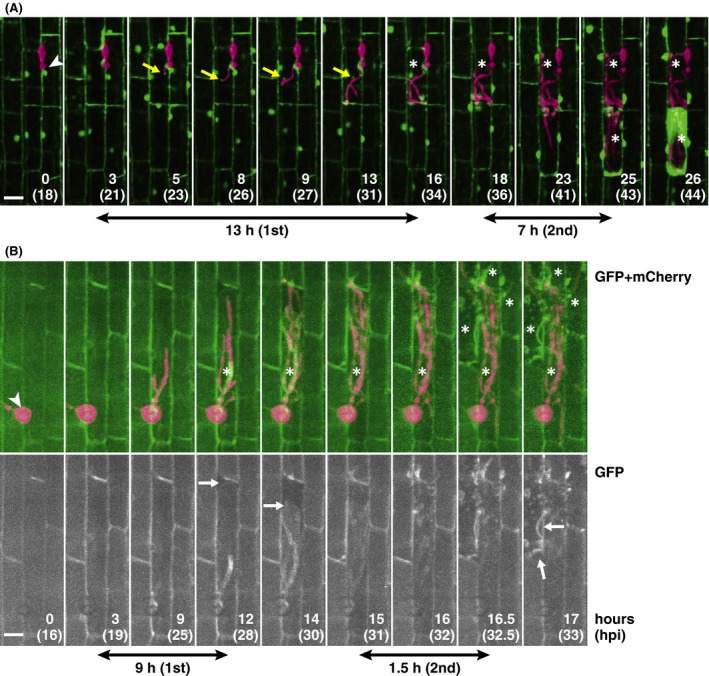
Time‐lapse imaging of a single infection site shows how the host central vacuole collapses and how long it takes. (A) Still images from Movie S2 showing host cytosol dynamics. Cyto‐GFP leaf sheaths were inoculated with the compatible TmC line. Yellow arrows indicate BIC and asterisks indicate the cells with shrinking or disrupted vacuole. (B) Still images from Movie S5 showing host vacuolar membrane dynamics. Vm‐GFP leaf sheaths were inoculated with the compatible TmC line. White arrows indicate the retracting host vacuole membrane and asterisks indicate the cells with the shrinking or disrupted vacuole. Double‐headed arrows indicate the approximate time from the start of fungal penetration to each cell to the start of cytosolic enlargement (A) or vacuolar shrinking (B). The cytosolic enlargement indicates vacuolar shrinking. Note that the shrinkage of host vacuoles commences earlier in the secondary‐invaded cells than in the primary‐invaded cells after the hyphal penetration. Each image is displayed with the elapsed time since the beginning of observation (upper numbers) and post inoculation in parentheses. Wedge, appressorium. Size bar = 20 *μ*m. GFP, green fluorescent protein; BIC, biotrophic interfacial complex.

Using this time‐lapse imaging system, we documented the dynamics of rice cytosol associated with the growth of invasive hyphae (Fig. 5A; Movies S2, S3). Because not all appressoria succeeded in colonization, a multipoint time‐lapse imaging mode was useful. The positional transition of BIC generated at the tip of the primary hyphae and then left behind was captured (Fig. 5A; Movie S2), confirming the biotrophic invasion process. As the invasive hyphae grew to reach the host cell end in the first‐invaded cell, cyto‐GFP signals were diffused and then attenuated. The enlargement of the cyto‐GFP area and its occupation of the whole‐cell volume were clearer in the next‐invaded cells (Fig. 5A; Movies S2, S3). These cytosolic dynamics were also demonstrated by monitoring the localization of a symplastic effector in nontransgenic rice tissues inoculated with the strain transformed with *PWL2p::GFP + PWL2p::PWL2:mCherry* (ProG+PWL2mC line) (Movie S4). The enlargement of the cyto‐GFP area and its occupation of the whole‐cell volume must reflect the shrink and collapse of the vacuole because the time‐lapse imaging of vm‐GFP signals confirmed these events (Fig. 5B; Movie S5). There was a duration of 2–3 h from the onset of vacuole shrinking to the collapse. Time after the hyphal invasion until the onset of vacuole shrinking was always longer in the first‐invaded cells than in the second‐invaded cells, although the time length varied by infection locus (Fig. 5; Movies S2, S3, S5). Altogether, the sequential recording of a single infection locus revealed that the host vacuoles gradually shrink to collapse; the vacuole collapse is initiated earlier in the neighboring cells, but the fungus continues to grow.

### Early vacuole disruption leads to hyphal lysis

Plant vacuoles contain several hydrolytic enzymes, including protease, chitinase, and *β*‐1,3‐glucanase. Vacuole collapse must involve the discharge of these contents, which may damage the invasive hyphae. However, the collapse of vacuoles appeared not to cause critical damage to the hyphae that had already developed to branch even in the first‐invaded cells because those hyphae continued to colonize the neighboring cells (Fig. 5; Movies S2, S3, S5). These observations prompted us to hypothesize that the timing of vacuole collapse is critical for determining the disruption effects on hyphal growth. Thus, we aimed to determine whether early collapse of host vacuoles involves the termination of the fungal growth in the compatible interaction. To assess if the invasive hyphae in a certain host cell with disrupted vacuoles show growth cessation, we focused on the leakage of fluorescent protein from invaded hyphae, which indicates fungal lysis. When rice plants were inoculated with an avirulent TmC line, mCherry signals were detected not only at invasive hyphae but also in the whole area of the invaded cell uniformly (Fig. S7A). This fluorescence pattern indicated that the hyphae were damaged to leak mCherry, and the host vacuoles collapsed. In the compatible interaction, mCherry signals were exclusively detected at the invasive hyphae even after the hyphae developed to branch (Fig. S7B), indicating that the fungus body was intact. However, even in the compatible combination, not all the appressoria resulted in the successful invasion, and some invaded cells showed a whole‐cell diffusion of mCherry signals though with low frequency. Thus, we inoculated cyto‐GFP plants with the virulent TmC line for simultaneously assessing the vacuole disruption and fungal lysis. Under an epifluorescence microscope, we searched the inoculated tissues at 30 hpi for the invaded cell showing the irregular fluorescence patterns: the dispersed or no cyto‐GFP signals and the leakage of mCherry signals from hyphae (Fig. S8). As shown in Table 2, in the three inoculation tests with five leaf sheaths each, we found 120 of the 3533 loci observed in total, exhibiting the irregular cyto‐GFP signal pattern, of which 98 loci (81.7%) showed the mCherry leakage. Similarly, 99 loci in total exhibited the irregular mCherry signals, of which 98 loci (99.0%) showed the irregular GFP signal pattern. In contrast, the ratio of infection loci with abnormal mCherry signals to normal GFP signals or that with abnormal GFP to normal mCherry signals were 0.03% and 0.60%, respectively (Table 2). These data showed a good correlation between the early collapse of vacuoles and damage of invasive hyphae and suggested that the successful colonization of the invasive hyphae in each infection locus in the host cell strongly depends on the maintenance of the host vacuole until a certain developmental stage.

**Table 2:** Correlation between cytoplasmic GFP signal patterns and mCherry leakage in the compatible interaction

GFP signals	Test	mCherry signals		
		Normal infection loci	Abnormal infection loci	Ratio (mCherry/GFP)
Normal	1	1282	0	
	2	1218	1	
	3	912	0	
	Total	3412	1	0.03%^a^
Abnormal	1	8	45	
	2	7	17	
	3	7	36	
	Total	22	98	81.7%^b^
	Ratio (GFP/mCherry)	0.60%^c^	99.0%^d^	

In each test, the sixth leaf sheath of five transgenic rice plants expressing AcGFP1 (cyto‐GFP line) were inoculated with a virulent strain expressing mCherry (TmC line) and the fluorescence patterns were evaluated for each appressorium (infection locus, 3533 loci in total) by microscopy at 30 hpi. GFP, green fluorescent protein.

a Abnormal mCherry/normal GFP.

b Abnormal mCherry/abnormal GFP.

c Abnormal GFP/normal mCherry.

d Abnormal GFP/abnormal mCherry.

## Discussion

### Close encasing of invasive hyphae by EIHM and the vacuolar membrane

Plant cells are characterized by a large central vacuole; thus, it is inevitable that filamentous pathogens growing inside the living host cell will share or compete for protoplasmic space with the vacuole. However, insights into the rice vacuoles during infection are lacking. In this study, we demonstrated that invasive hyphae of *M. oryzae* invaginate, but do not penetrate across the vacuolar membrane; in the compatible interaction, the invasive hyphae are surrounded by the vacuolar membrane even after they become highly branched (Fig. 2A). Situation in the incompatible interaction was the same before hypersensitive cell death occurred (Fig. S2). Vm‐GFP and Bas4:mCherry signals were similarly detected, outlining the primary hyphae and bulbous invasive hyphae (Fig. 3A), whereas cyto‐GFP and er‐GFP were not detected along the invasive hyphae that had grown bulbous (Fig. 1B; Fig. S1A). These observations indicate that the vacuolar membrane very closely surrounds the bulbous invasive hyphae that are encased by EIHM. Bas4:mCherry signals around the hyphae remained even after vm‐GFP signals disappeared, and both signals did not always overlap (Fig. S3), indicating that the vacuolar membrane that encases the invasive hyphae is different from EIHM. The initiation of vacuole shrinking was a sign of lost integrity, which was probably caused by the hyphal penetration across the vacuolar membrane (Fig. 2B). Both vacuolar membranes and EIHM appeared to lose integrity during the same infection stage (Fig. 3B; Fig. S3A), although it is unknown which membrane was broken earlier.

EIHM is considered to have originated from the host plasma membrane, as indicated by the observation of transgenic rice lines expressing GFP‐LTi6b (Mentlak et al. 2012). GFP‐LTi6b was isolated as a marker for the plasma membrane of Arabidopsis by a random GFP:cDNA fusion approach (Cutler et al. 2000). We also confirmed that GFP‐LTi6b is a useful marker for visualizing the EIHM encasing the bulbous invasive hyphae in the experimental conditions of this study (Fig. S4). In contrast, we have recently reported that a GFP‐tagged plasma membrane‐resident protein, OsCERK1 (a pattern recognition receptor for chitin oligomers; Shimizu et al. 2010), is detected around the primary hyphae as vesicle‐like bodies, but not encasing the bulbous invasive hyphae (Kouzai et al. 2014), unlike GFP‐LTi6b. We examined another plasma membrane‐localizing rice protein, EL5, which is an ubiquitin‐ligase expressed in response to the chitin oligomers (Takai et al. 2002). The signals of EL5∆24‐GFP, which is a stabilized mutant EL5 (Koiwai et al. 2007), were detected to encase only the primary hyphae and occasionally BIC‐associated first bulbous cell (Fig. S9). These data indicate that a part of the plasma membrane‐localizing proteins are excluded from EIHM as hyphae grow; thus, the components of EIHM are different from the plasma membrane, although it appears as an invagination from the plasma membrane. It has been shown that numerous host plasma membrane proteins, including pattern recognition receptors, are absent from EHM in Arabidopsis infected with the powdery mildew fungus (Koh et al. 2005; Micali et al. 2011) or oomycetes, *H. arabidopsidis* and *Phytophthora infestans* (Caillaud et al. 2012; Lu et al. 2012), and suggested that EHM originates from the de novo assembly of plant membranes or remodeling of the existing plasma membrane with the exclusion of host proteins. The exclusion of defense‐related proteins, such as the pattern recognition receptors from EIHM or EHM, could be one mechanism employed by pathogens to avoid detection by the host immune system. However, currently, we speculate that close proximity between EIHM and the vacuolar membrane around the developed invasive hyphae obstructs the vesicle trafficking to supply OsCERK1 and EL5 toward EIHM, and only certain stable proteins, such as GFP‐LTi6b, enable the continuous labeling of EIHM.

The large surface area of vacuole membranes and EIHM must be constructed as the invasive hyphae grow. The fungus may redirect membrane trafficking in the host cell for supplying membrane components for building the vacuolar membrane, as proposed for EIHM (Kankanala et al. 2007). Future research will be required to address how host membranes are generated and how *M. oryzae* controls the membrane dynamics during the biotrophic invasion.

### Timing of vacuole collapse and virulence

In this study, we developed a method for long‐term time‐lapse fluorescence imaging with sliced inner epidermal sections of leaf sheaths, by which we monitored growing invasive hyphae (labeled with mCherry) and host cytosol or vacuolar membranes (labeled with GFP) simultaneously, three‐dimensional, and sequentially over 40 h in 20‐ or 30‐min intervals. Although the frequency of successful infection was lower than the conventional fixed time point observations using leaf sheath tissues mainly because of the frequent laser radiation, the long‐term time‐lapse imaging method enabled to capture the biotrophic invasion process and multicell colonization, which were comparable to infection observed at the fixed time point (Figs. 1, 2, 3).

The time‐lapse imaging has documented that the vacuoles in the first‐invaded cells gradually shrink to collapse at the time when invasive hyphae are about to cross the inner cell walls (Fig. 5; Movies S2–S5). The collapse of the central vacuole may damage fungal growth because of the discharge of antifungal compounds, such as chitinase. Even in the compatible interaction, not all fungal penetration succeeded in colonizing rice cells, and a good correlation was observed between the early collapse of vacuoles and damage of the invasive hyphae (Table 2); 81.7% of infection loci revealing irregular cyto‐GFP signals vacuolar disruption at the early infection stage showed mCherry leakage from the invasive hyphae. Viewed from another angle, 99.0% of infection loci revealing the hyphal lysis indicated the vacuolar disruption. These data strongly suggest that maintenance of host vacuoles until a certain stage, probably when the invasive hyphae develop to highly branched, is necessary for *M. oryzae* to establish the biotrophic invasion of rice cells.

The rice blast fungus modifies the cell wall structure of the invasive hyphae. For example, *α*‐1,3‐glucan is produced in an infection‐dependent manner to mask the surface chitin and minimize the attack by host chitinase (Fujikawa et al. 2009), suggesting a fungal mechanism for providing defense against the ultimate collapse of vacuoles and the subsequent discharge of lytic enzymes. Kankanala et al. (2007) has reported that a time‐dependent switch occurred during the growth in the first‐invaded cells because invasive hyphae tended to spread into neighboring cells between 32 and 36 hpi, no matter how quickly or completely, the first cell had been filled with fungus. Our observations strongly suggested that host vacuoles collapse around the time when the time‐dependent switch occurs; thus, the switch may correspond with the stage when the fungus acquires the tolerance to vacuole collapse or host cell death.

In contrast, the time‐lapse imaging showed that the vacuolar collapse before the hyphal branching appeared to not affect the fungal growth in the secondary‐invaded cells (Fig. 5; Movies S2, S3, S5). It has been indicated that the invasive hyphae in the secondary and subsequently invaded rice cells differ in behavior from the invasive hyphae in the primary‐invaded cell; bulbous invasive hyphae in subsequently invaded cells grow more rapidly from one cell to another (Kankanala et al. 2007). Interestingly, chitin exposure also differs in the secondary‐invaded cells; invasive hyphae in the primary‐invaded cells showed only weak staining with a fluorescent probe to detect chitin; however, once the invasive hyphae entered the neighboring cells, they demonstrated higher chitin exposure and the fluorescent signals lasted for a longer time (Mochizuki et al. 2011). These data strongly suggest that the vacuole collapse during early infection stages (before branching in the first‐invaded cells) is critical to the hyphal growth, and *M. oryzae* may actively suppress the collapse but subsequently, when the invasive hyphae colonize the next cells, the fungus acquires tolerance to the host vacuole collapse. Host immunity in the neighboring cells may be decreased by fungal effectors prior to the fungal invasion.

The mechanism used to avoid host vacuole collapse during pathogenesis is unknown. An effector of *H. arabidopsidis*, HaRxL17, has been recently shown to associate with the vacuolar membrane in uninfected cells and probably with EHM in infected cells and confer enhanced plant susceptibility when expressed in Arabidopsis (Caillaud et al. 2012). Although any blast fungus effectors showing host membrane localization have not been identified yet, some effectors may contribute to the pathogenicity by stabilizing the host vacuolar membrane.

### High‐resolution imaging of BIC

BIC was initially identified as a membranous cap at the tip of primary hyphae and the filamentous invasive hyphae that crossed to neighboring cells (Kankanala et al. 2007) and was later defined as a pathogen‐induced plant‐derived structure, where symplastic effectors preferentially localize (Khang et al. 2010; Giraldo et al. 2013). We demonstrated by high‐resolution imaging that highly aggregated host plasma membrane/EIHM with cytosol forms the BIC structure, whereas the vacuolar membrane does not comprise but closely encloses BIC (Fig. 4). Host cytosol appeared to be entangled in the aggregated EIHM but did not surround it. Furthermore, a symplastic effector, Pwl2, has been shown to exist in the BIC in a punctate form (Fig. 4; Fig. S5). Knowledge of BIC strongly suggests a central role for BIC as a destination for secreted effectors that are ultimately taken up by rice cells (Giraldo et al. 2013). Although further studies are required for elucidating how symplastic effectors are translocated into the host cell, our observation implies the involvement of membrane fusion in the effector internalization.

Our imaging data also demonstrated that Bas4 is an excellent marker for simultaneous imaging of BIC position and EIHM as well as for assessing EIHM compartment integrity (Mosquera et al. 2009). Bas4:mCherry showed substantial accumulation in the periphery of the BIC‐associated cell at the base of BIC, and the accumulation area of Bas4:mCherry at the BIC was always smaller than that of Pwl2:mCherry (Fig. 4). These observations suggest that the BIC comprises the region reachable for both apoplastic and symplastic effectors (the basement of BIC) and the region where only symplastic effectors can reach (detected as puncta in the BIC). Symplastic effectors are also detected in EIHMx around the BIC‐associated cells (primary hyphae and first‐differentiated bulbous invasive hyphal cell) when images are gained with increased exposure (our data not shown and Khang et al. 2010); thus, an unknown mechanism should be employed for facilitating the preferential localization of symplastic effectors at the base of BIC, which might trap apoplastic effectors partially.

## Conflict of Interest

None declared.

## Supporting information


**Figure S1.** Primary invasive hyphae of *Magnaporthe oryzae* are surrounded by host ER. Leaf sheaths of transgenic rice plants constitutively expressing EGFP:HDEL (er‐GFP line) were inoculated with a conidial suspension of a compatible strain transformed with *tefp::mCherry* (TmC1 line) and observed using a laser confocal microscope. GFP, stacked *z*‐series confocal fluorescence images of GFP signals corresponding roughly to the surface half of rice epidermal cells. mCherry + GFP, mergers of the GFP image, and stacked *z*‐series confocal fluorescence images of mCherry signals. DIC, differential interference contrast images. Yellow arrows in the B (GFP) indicate the invasive hyphae surrounded by ER in the second‐invaded cells. Wedge, appressorium. Size bar = 20 *μ*m.
**Figure S2.** Subcellular changes in host cells in the incompatible interaction. Leaf sheaths of transgenic rice plants with fluorescently labeled cytosol (cyto‐GFP), ER (er‐GFP), and vacuolar membranes (vm‐GFP) were inoculated with a conidial suspension of an incompatible strain (P91‐15B) transformed with *tefp::mCherry*, and observed using a laser confocal microscope at 30 hpi. Representative data of 3 (cyto‐GFP), 3 (er‐GFP), and 8 (vm‐GFP) similar images are shown. GFP, stacked *z*‐series confocal fluorescence images of GFP signals corresponding roughly to the surface half of rice epidermal cells. mCherry + GFP, mergers of the GFP image, and stacked *z*‐series confocal fluorescence images of mCherry signals. DIC, differential interference contrast images. Arrows indicate the invasive hyphae outlined by GFP signals. The presented data show that the invasive hyphae are surrounded by host vacuole even in the incompatible interaction before the hypersensitive cell death occurs. Wedge, appressorial penetration site. Size bar = 20 *μ*m.
**Figure S3.** Extrainvasive hyphal membrane (EIHM) in the host cell with the damaged vacuole. Rice leaf sheaths with GFP‐labeled vacuolar membranes (vm‐GFP line) were inoculated with a conidial suspension of a compatible strain transformed with *BAS4p::BAS4:mCherry* (BAS4mC line) and observed using a laser confocal microscope. Vm‐GFP signals indicate the vacuolar shrinkage in (A) and collapse in (B). Bas4:mCherry signals are patchy, indicating that EIHM lost integrity. GFP, stacked *z*‐series confocal fluorescence images. mCherry + GFP, mergers of the GFP image, and stacked *z*‐series confocal fluorescence images of mCherry signals. DIC, differential interference contrast images. Wedge, appressorium. Size bar = 20 *μ*m.
**Figure S4.** Confirmation that extrainvasive hyphal membrane (EIHM) is visualized by using a host plasma membrane‐marker protein, EGFP:LTi6b. Leaf sheaths of transgenic rice plants constitutively expressing EGFP:LTi6b (pm‐GFP line) were inoculated with a conidial suspension of a compatible strain transformed with *BAS4p::BAS4:mCherry* (BAS4mC line) and observed using a laser confocal microscope at 30 hpi. Wedge, appressorium. Size bar = 20 *μ*m.
**Figure S5.** Pwl2 accumulates in BIC in a punctated form. Leaf sheaths were inoculated with a conidial suspension of a compatible strain transformed with *PWL2p::PWL2:mCherry* (PWL2mC line) and observed using a laser confocal microscope at 30 hpi. Representative PWL2:mCherry signals in the typical BICs of 15 images are shown with bright field images (BF). Size bar = 1 *μ*m.
**Figure S6.** Method of sample preparation for long‐term live‐cell imaging during early infection stages of *Magnaporthe oryzae*. (A) Schematic diagram of a transverse section of rice leaf sheath. The inner epidermal cell layer, including 2–3 cell layers of parenchyma (ca. 60–80 *μ*m in thickness; encircled with the red broken line) was cut off with a razor blade and inoculated with a conidial suspension of *M. oryzae*. (B) A unit of the prepared specimen for long‐term time‐lapse imaging. (C) Scheme of time‐lapse fluorescence imaging.
**Figure S7.** Fluorescence signal diffused in the invaded rice cell is confirmed as hyphal protein leakage. Leaf sheaths of rice plants with (A) and without (B) *Pik*, a blast‐disease resistant gene, were inoculate with a strain (Ina86‐137), which possesses *AVR‐Pik,* transformed with *tefp::mCherry* (TmC line). Fluorescence excited with an argon laser at 561 nm was scanned in 10‐nm wavelength widths from 580 to 620 nm at 5‐nm intervals. Mean intensity of the 12‐bit gray scale value from each region shown in the left fluorescence images was graphed. The spectral pattern of mCherry was obtained from the invasive hypha (red regions) as control. Size bar = 20 *μ*m.
**Figure S8.** Representative confocal images of the infection loci with the irregular GFP and mCherry signal patterns. (A) Infection locus with the dispersed cyto‐GFP signals, which indicates the shrinkage of the central vacuole, and the leakage of mCherry from the invasive hyphae. (B) Infection locus showing no cyto‐GFP signals, which indicates vacuole collapse or cell death, and the mCherry leakage. 30 hpi. Size bar = 20 *μ*m.
**Figure S9.** Localization of a rice plasma membrane‐resident protein, EL5, in the infected cell. Leaf sheaths of transgenic rice plants constitutively expressing EL5∆24:GFP were inoculated with a conidial suspension of a compatible strain transformed with *BAS4p::BAS4:mCherry* (BAS4mC line) to visualize the EIHMx and observed using a laser confocal microscope at 30 hpi. *EL5* used was mutated for stabilizing the protein because the authentic EL5 is too unstable to be detected. Wedge, appressorium. Size bar = 20 *μ*m.
**Figure S10.** Confirmation of the specific labeling of the subcellular structures in transgenic rice plants. Fluorescence images of leaf sheath cells of transgenic plants with GFP‐labeled cytosol (cyto‐GFP), endoplasmic reticulum (er‐GFP), vacuolar membrane (vm‐GFP), and plasma membrane (pm‐GFP). Size bar = 20 *μ*m.

**Table S1.** Oligonucleotide primers used in this work.

**Movie S1.** Long‐term fluorescence imaging of invasive growth of *Magnaporthe oryzae* in rice cells. Leaf sheaths were inoculated with a conidial suspension of a compatible strain transformed with *tefp::mCherry* (TmC line), and 97 images in total were acquired from 18 hpi for 48 h at every 30 min. Each frame of the movie is stacked *z*‐series confocal fluorescence images. Size bar = 20 *μ*m.

**Movie S2.** Host cytosolic dynamics during biotrophic invasion by *Magnaporthe oryzae* (1). Time‐lapse imaging was conducted from 18 hpi for 30 h, with a total 91 images acquired every 20 min. A conidial suspension of *M. oryzae* expressing mCherry (TmC line) was inoculated on the leaf sheath expressing AcGFP1 (cyto‐GFP line). Stacked *z*‐series confocal fluorescence images for mCherry (left), GFP (middle), and merged images (right) are sequentially shown. Several frames are shown in Figure 5A. Size bar = 20 *μ*m.

**Movie S3.** Host cytosolic dynamics during biotrophic invasion by *Magnaporthe oryzae* (2). Time‐lapse imaging was conducted from 18 hpi for 19.5 h, with a total 40 images acquired every 30 min. A conidial suspension of *M. oryzae* expressing mCherry (TmC line) was inoculated on the leaf sheath expressing AcGFP1 (cyto‐GFP line). Stacked *z*‐series confocal fluorescence images for mCherry (left), GFP (middle), and the merged images (right) are sequentially shown. Size bar = 20 *μ*m.

**Movie S4.** Dynamics of Pwl2 localization in the host cells. Time‐lapse imaging was conducted from 22 hpi for 15.5 h, with a total 32 images acquired every 30 min. A conidial suspension of *M. oryzae* harboring *PWL2p::GFP* and *PWL2p::PWL2:mCherry* (ProG+PWL2mC line) was inoculated on rice leaf sheaths. *Z*‐series of confocal fluorescence images for GFP (indicating *PWL2* expression) and mCherry (indicating the Pwl2 localization in host cytosol) was superimposed. Size bar = 20 *μ*m.

**Movie S5.** Sequential imaging of shrink and collapse of host vacuolar membranes during biotrophic invasion by *Magnaporthe oryzae*. Time‐lapse imaging was conducted from 16 hpi for 24.5 h, with a total 50 images acquired every 30 min. A conidial suspension of *M. oryzae* expressing mCherry (TmC line) was inoculated on the rice leaf sheaths with GFP‐labeled vacuolar membranes (vm‐GFP line). Stacked *z*‐series confocal fluorescence images for mCherry (left), GFP (middle), and the merged images (right) are sequentially shown. Several frames were selected and are shown in Figure 5B. Size bar = 20 *μ*m.
