# Supplementary material for: Live‐cell imaging of rice cytological changes reveals the importance of host vacuole maintenance for biotrophic invasion by blast fungus, Magnaporthe oryzae
Source: Microbiologyopen. 2015 Oct 15;4(6):952–66. doi: 10.1002/mbo3.304 (PMC4694143; doi:10.1002/mbo3.304)

Fig. S1

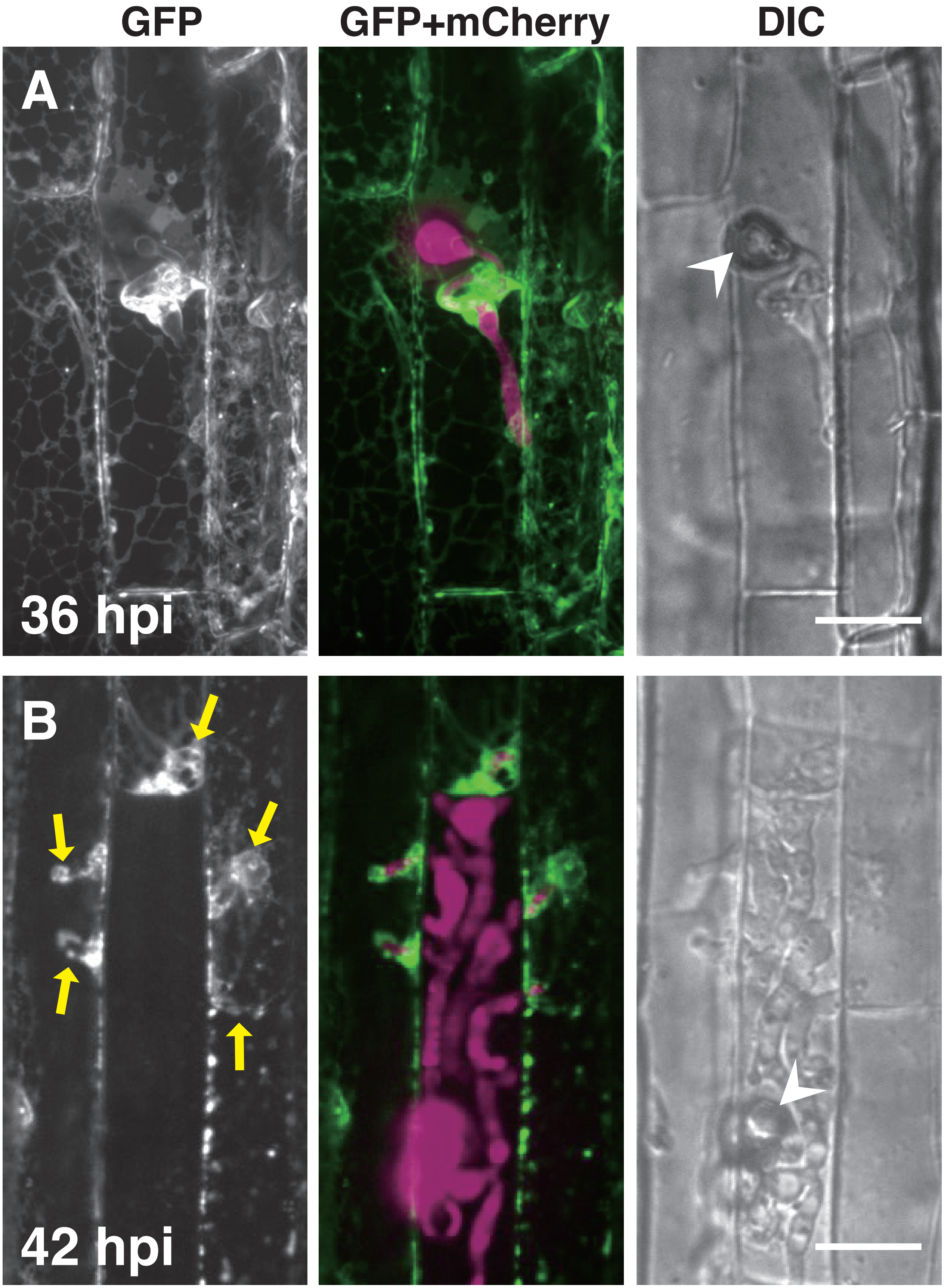

Fig. S2

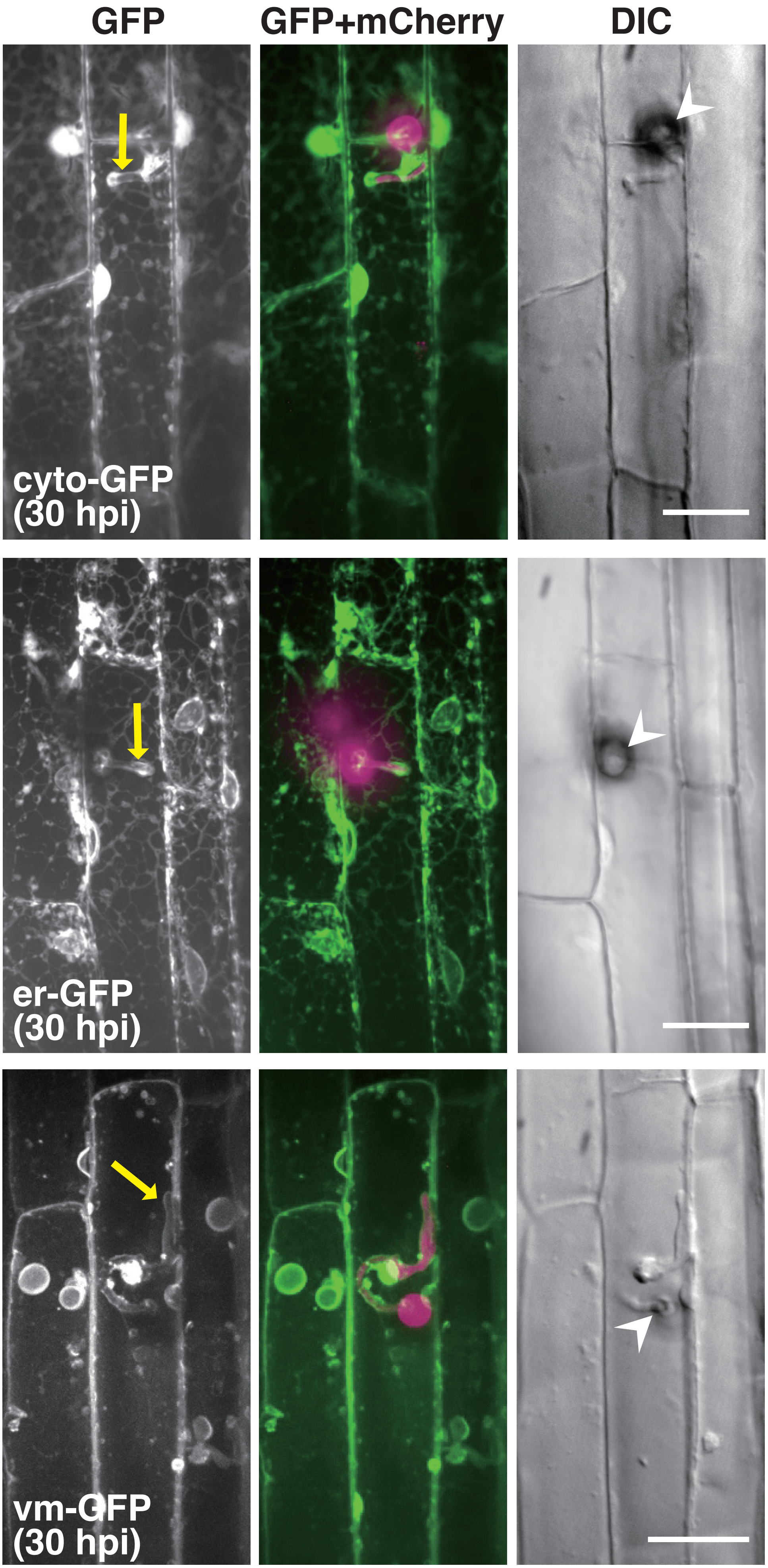

Fig. S3

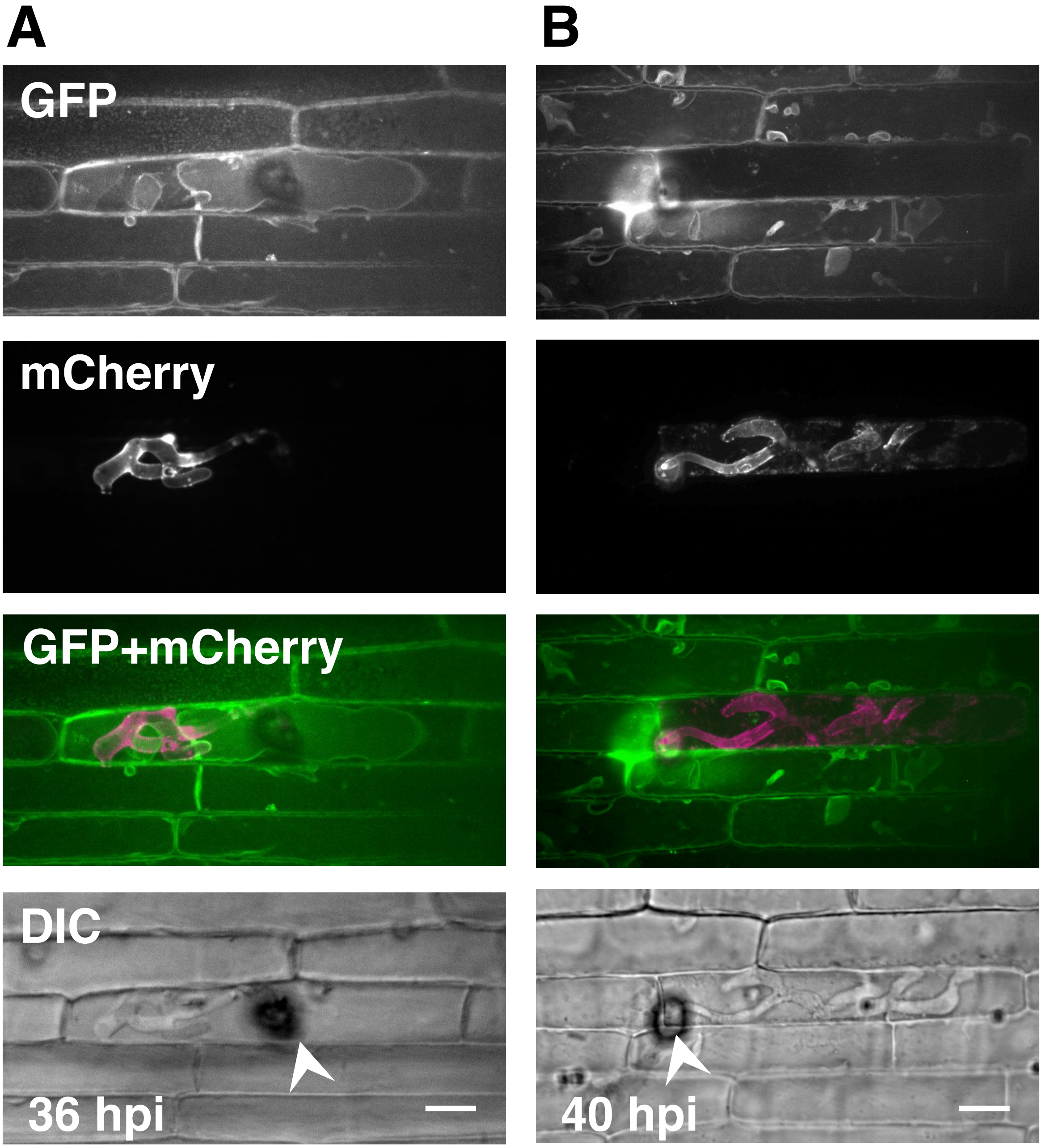

Fig. S4

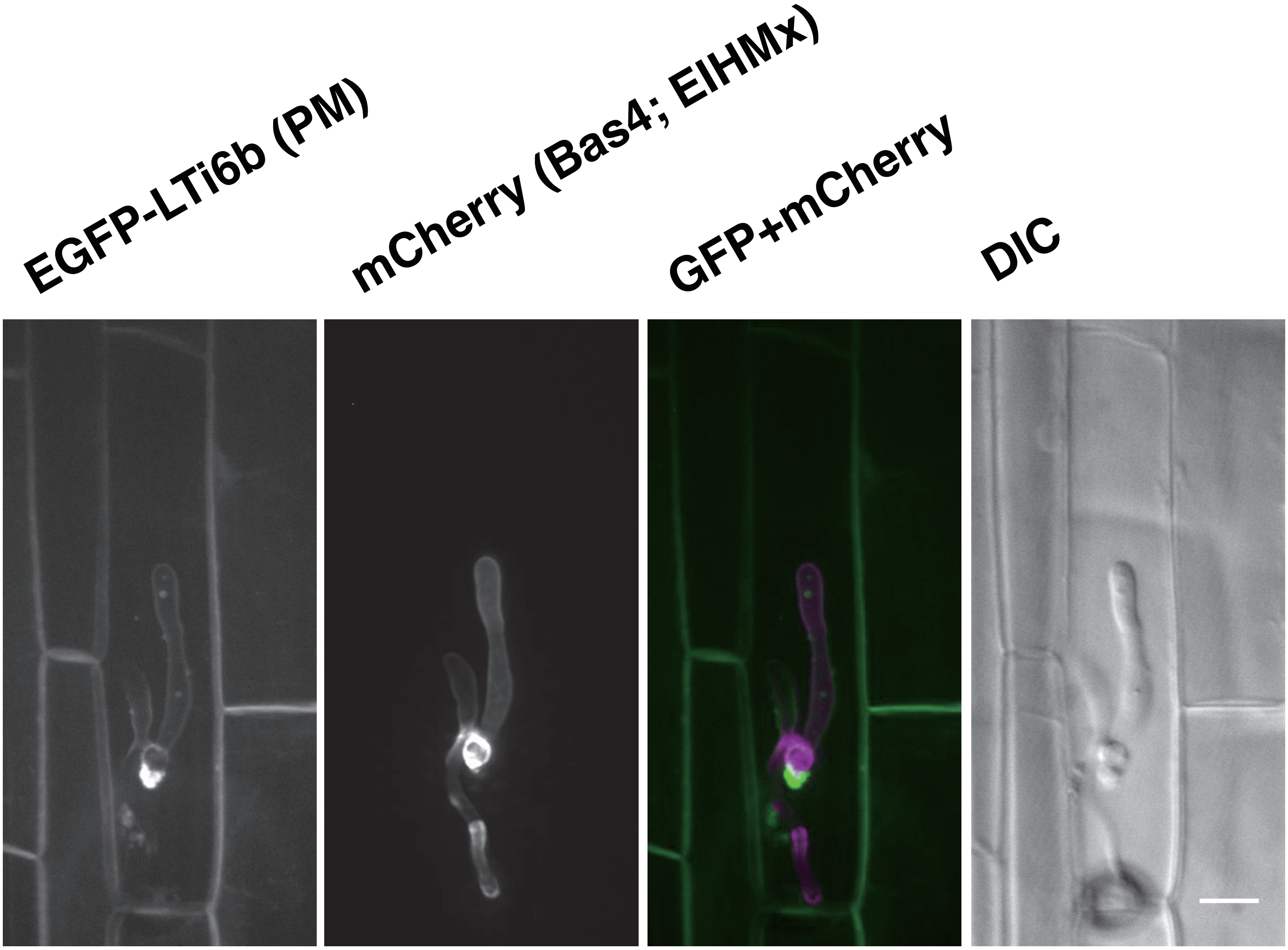

Fig. S5

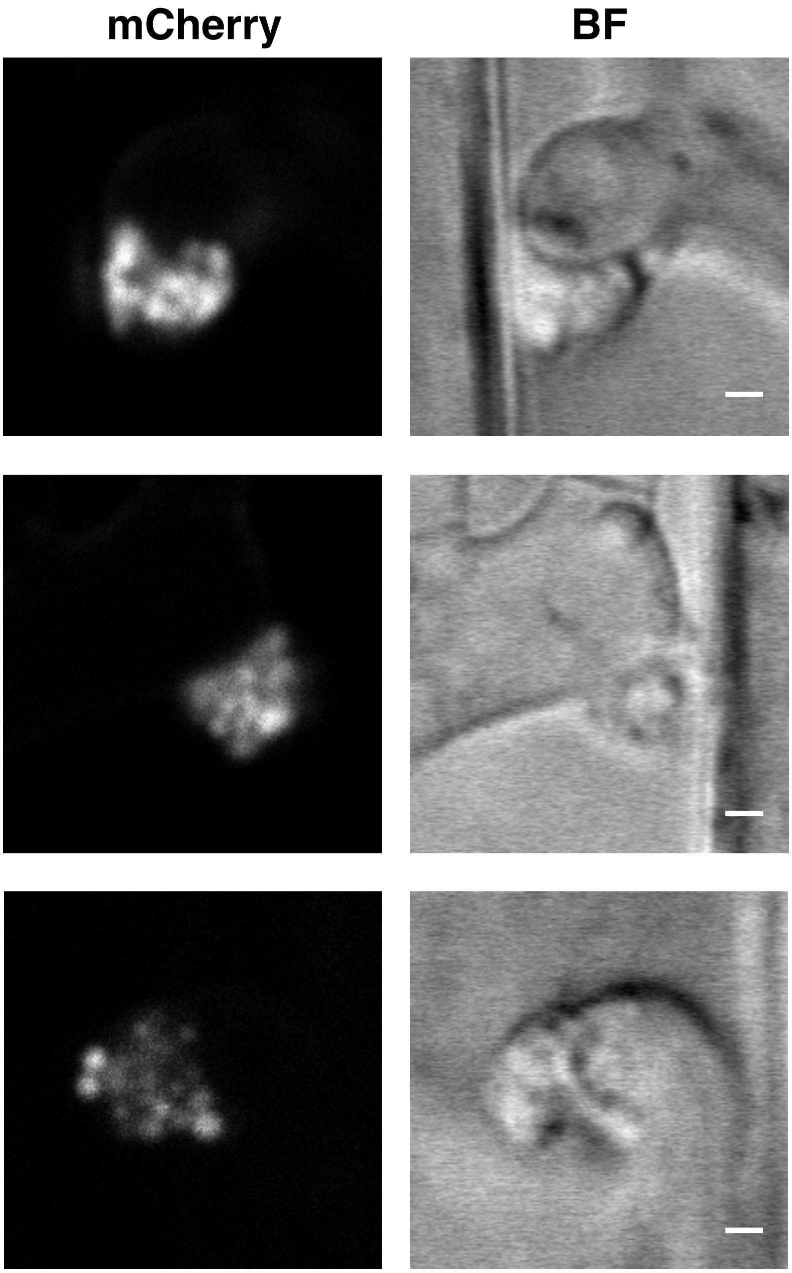

Fig. S6

A

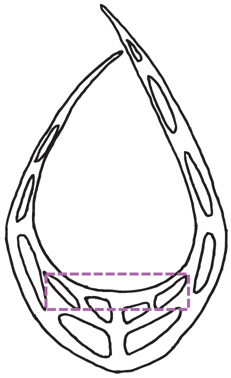

B

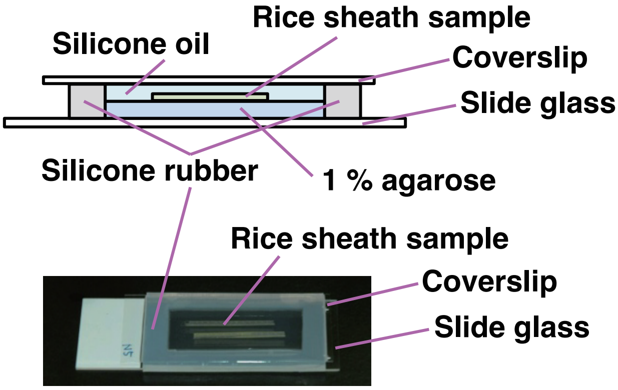

C

Collect sliced-leaf-sheath (3-cm length)
from the 6th leaf

⬇

Tame sliced-leaf-sheath for 2-h on water

⬇

Pour low-melting agarose in PBS (pH 7.4)
into the space surrounded by silicone spacer
(1.5 cm × 4.0 cm × 3 mm) put on a slide glass

⬇

Mount the tamed leaf-sheath on the agarose

⬇

Incubate in dark & moist chamber for 1 h

⬇

Put conidial suspension
(5×10^5 conidia ml^{-1}; 200 *μ*l)
on the leaf sheaths

⬇

Incubate at 25 °C in dark & moist chamber for 12 h

⬇

(Check the success in fungal infection)
Fill the top space with silicone oil
[dimethylpolysiloxane (200 cSt)]
and cover with a coverslip

⬇

Collect fluorescence images using a confocal
laser scanning microscope in the room at 25 °C

Fig. S7

A

Incompatible combination

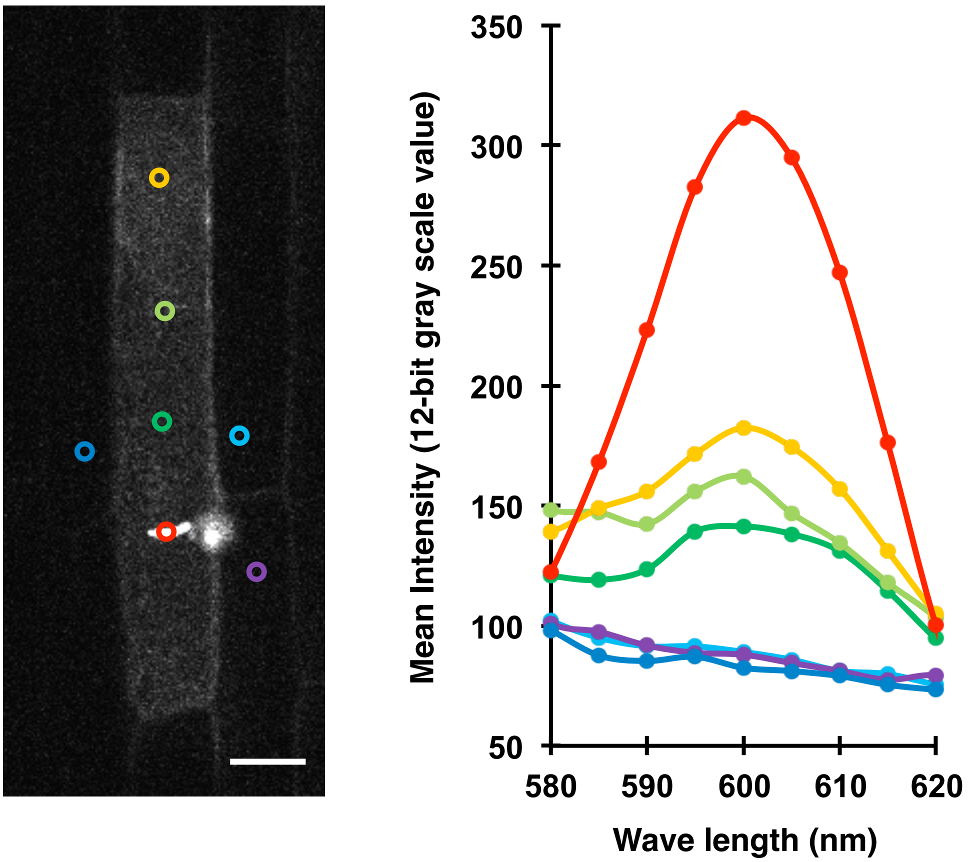

B

Compatible combination

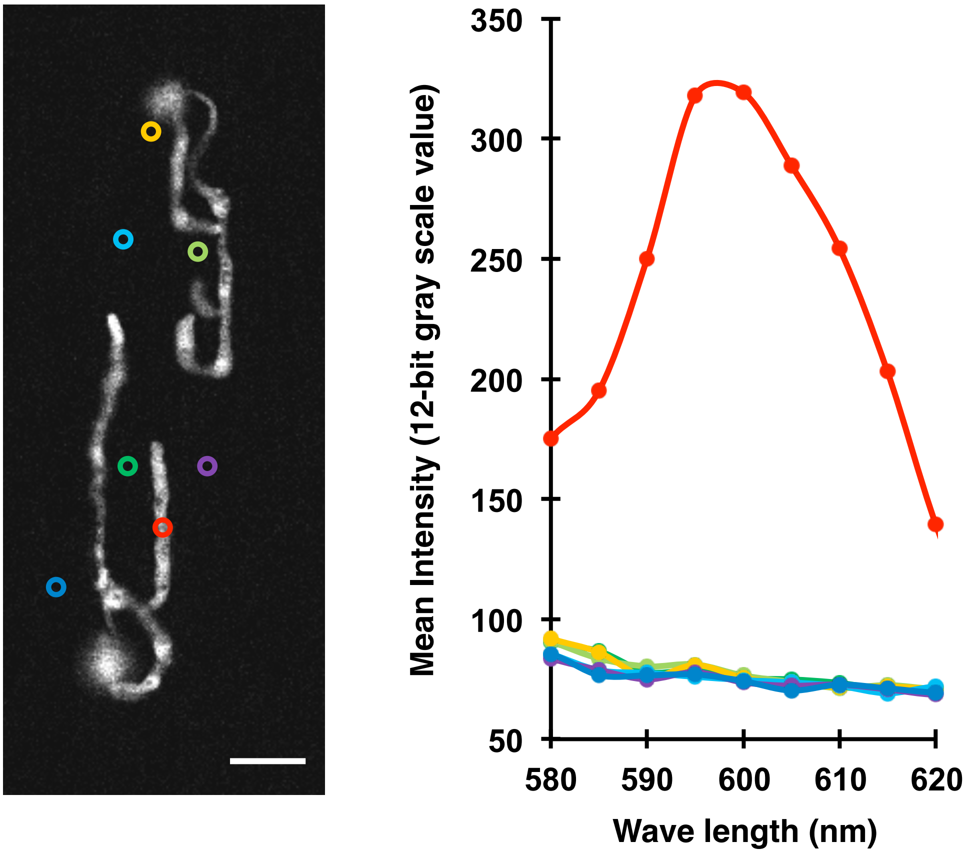

Fig. S8

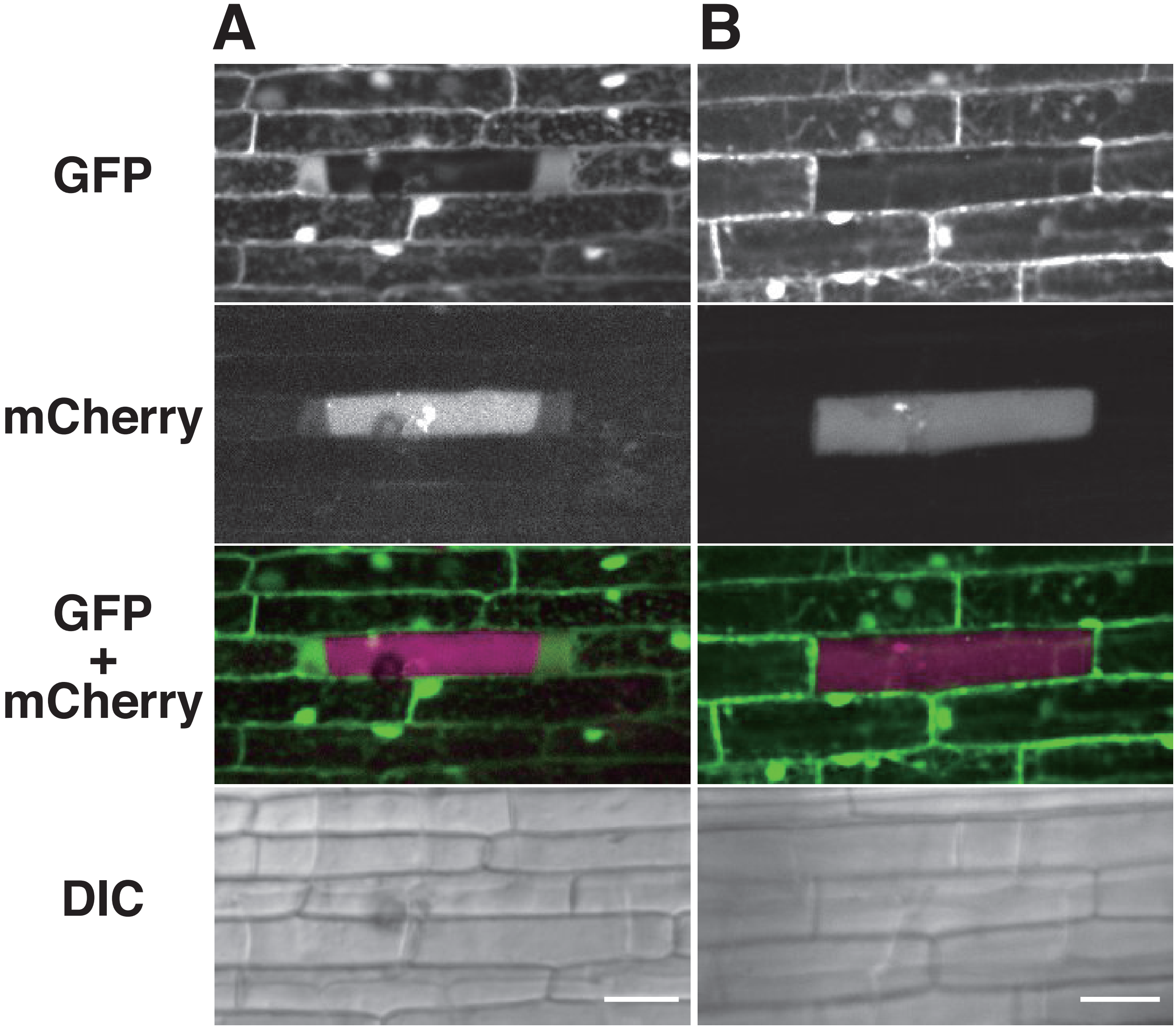

Fig. S9

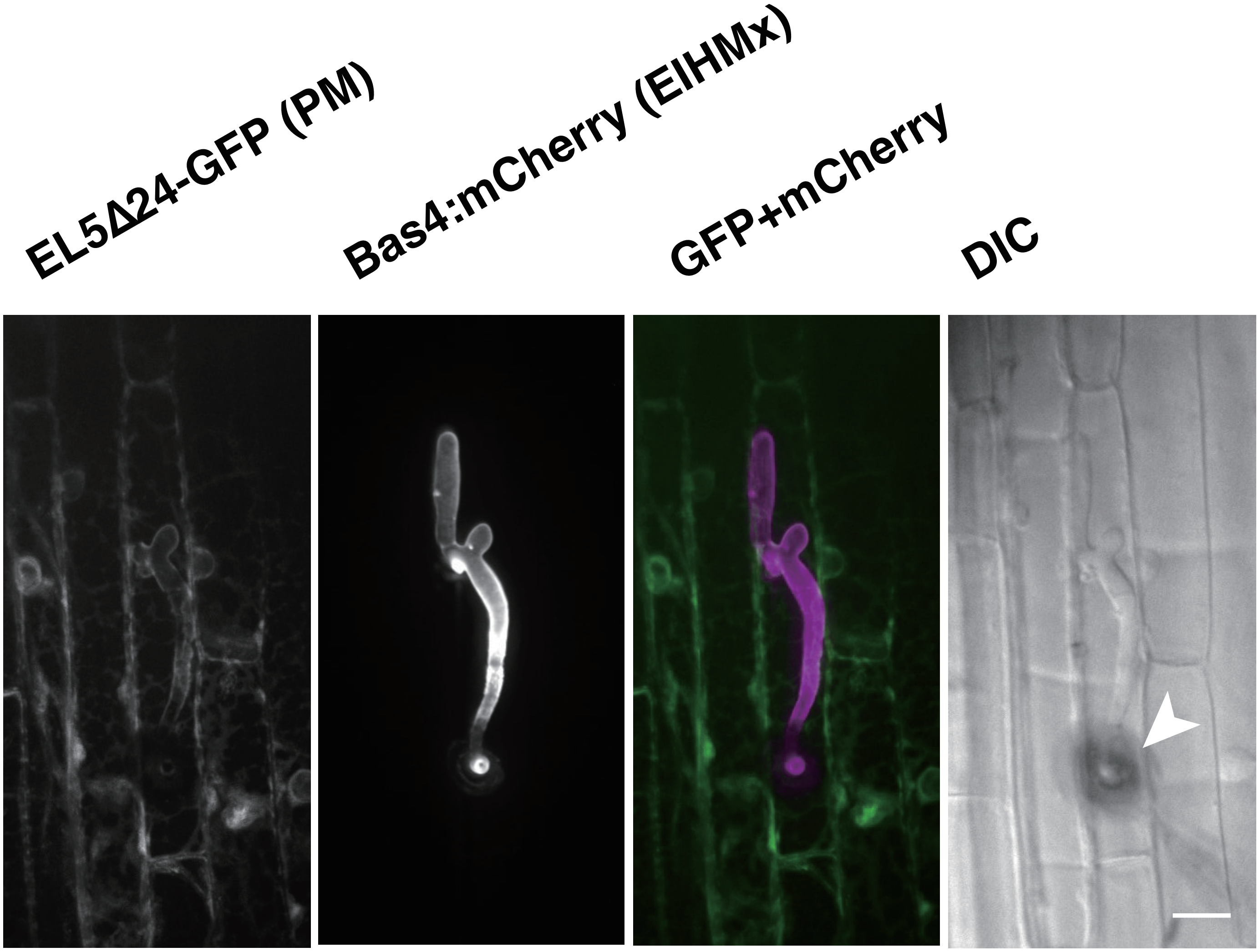

Fig. S10

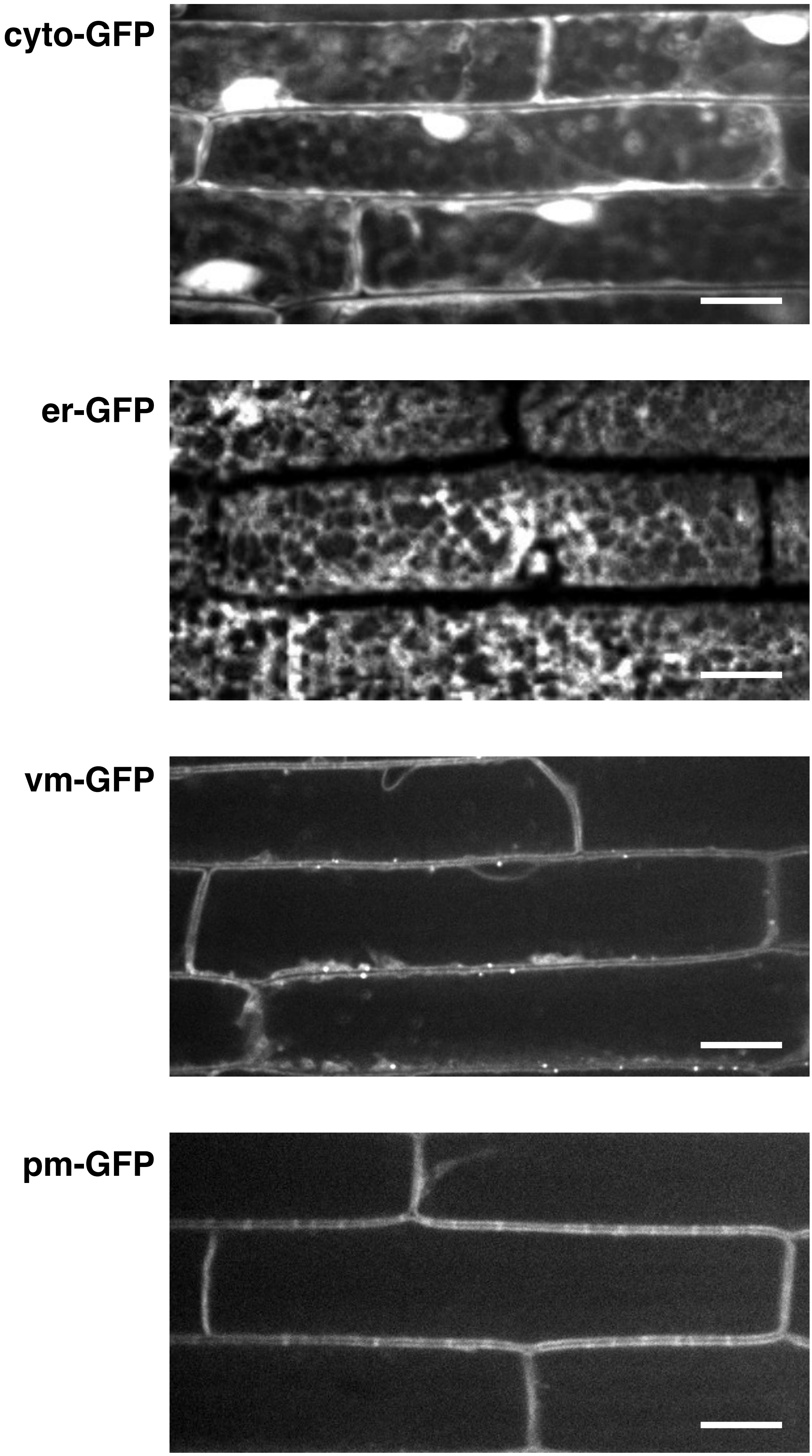

Supplement: Supplementary file 1 — Figure S1. Primary invasive hyphae of Magnaporthe oryzae are surrounded by host ER. Leaf sheaths of transgenic rice plants constitutively expressing EGFP:HDEL (er‐GFP line) were inoculated with a conidial suspension of a compatible strain transformed with tefp::mCherry (TmC1 line) and observed using a laser confocal microscope. GFP, stacked z‐series confocal fluorescence images of GFP signals corresponding roughly to the surface half of rice epidermal cells. mCherry + GFP, mergers of the GFP image, and stacked z‐series confocal fluorescence images of mCherry signals. DIC, differential interference contrast images. Yellow arrows in the B (GFP) indicate the invasive hyphae surrounded by ER in the second‐invaded cells. Wedge, appressorium. Size bar = 20 μm. Figure S2. Subcellular changes in host cells in the incompatible interaction. Leaf sheaths of transgenic rice plants with fluorescently labeled cytosol (cyto‐GFP), ER (er‐GFP), and vacuolar membranes (vm‐GFP) were inoculated with a conidial suspension of an incompatible strain (P91‐15B) transformed with tefp::mCherry, and observed using a laser confocal microscope at 30 hpi. Representative data of 3 (cyto‐GFP), 3 (er‐GFP), and 8 (vm‐GFP) similar images are shown. GFP, stacked z‐series confocal fluorescence images of GFP signals corresponding roughly to the surface half of rice epidermal cells. mCherry + GFP, mergers of the GFP image, and stacked z‐series confocal fluorescence images of mCherry signals. DIC, differential interference contrast images. Arrows indicate the invasive hyphae outlined by GFP signals. The presented data show that the invasive hyphae are surrounded by host vacuole even in the incompatible interaction before the hypersensitive cell death occurs. Wedge, appressorial penetration site. Size bar = 20 μm. Figure S3. Extrainvasive hyphal membrane (EIHM) in the host cell with the damaged vacuole. Rice leaf sheaths with GFP‐labeled vacuolar membranes (vm‐GFP line) were inoculated with a conidial suspe [file MBO3-4-0952-s001.pdf]
